# Application of artificial intelligence in mine ventilation: a brief review

**DOI:** 10.3389/frai.2024.1402555

**Published:** 2024-05-02

**Authors:** Mikhail Semin, Denis Kormshchikov

**Affiliations:** Mining Institute of the Ural Branch of the Russian Academy of Sciences, Perm, Russia

**Keywords:** mine ventilation, artificial intelligence, air distribution, monitoring systems, ventilation faults, underground fires, shock losses

## Abstract

In recent years, there has been a notable integration of artificial intelligence (AI) technologies into mine ventilation systems. A mine ventilation network presents a complex system with numerous interconnected processes, some of which pose challenges for deterministic simulation methods. The utilization of machine learning techniques and evolutionary algorithms offers a promising avenue to address these complexities, resulting in enhanced monitoring and control of air parameter distribution within the ventilation network. These methods facilitate the timely identification of resistance faults and enable prompt calculation of ventilation parameters during emergency scenarios, such as underground explosions and fires. Furthermore, evolutionary algorithms play a crucial role in the advancement of methods for visual analysis of ventilation systems. However, it is essential to acknowledge that the current utilization of AI technologies in mine ventilation is limited and does not encompass the full spectrum of challenging-to-formalize problems. Promising areas for AI application include analyzing changes in air distribution caused by unaccounted thermal draft and gas pressure, as well as developing novel approaches for calculating shock losses. Moreover, the application of AI technologies in optimizing large-scale mine ventilation networks remains an unresolved issue. Addressing these challenges holds significant potential for enhancing safety and efficiency in mine ventilation systems.

## 1 Introduction

The ventilation system is an important element for the trouble-free operation of a mine and is often described as the respiratory system (Liu et al., 2022a) or the lifeblood of a mine (McPherson, 2012). Its task is to continuously supply fresh air to all underground working areas to dilute and remove various harmful impurities such as gas and dust, while also providing comfortable microclimatic conditions determined by air velocity, temperature, and relative humidity. The need to organize an effective ventilation system in mines is reinforced by specific requirements in regulatory documents of many countries (Semin et al., 2020).

The consumption of mineral resources is constantly growing, accompanied by a continual increase in mining capacity. Mines are expanding and branching out, with underground mining being carried out using increasingly high-performance equipment (Jia et al., 2020). The amount of harmful emissions released during mining operations is increasing, and the task of delivering the required amount of air to working areas is becoming more challenging for mining enterprises (Shriwas and Pritchard, 2020).

Complex mine ventilation network analysis relies heavily on analytical tools and software systems. Improving ventilation necessitates numerical simulation to manage airflows and pollutants (Yi et al., 2022). Yet, mine ventilation networks are intricate, with numerous degrees of freedom (Liu et al., 2022a). Constructing mathematical models for such networks proves challenging due to substantial input data errors from field measurements and unpredictable aerodynamic factors. This compels engineers and scientists to seek novel approaches to ventilation issues.

**Figure 1:**
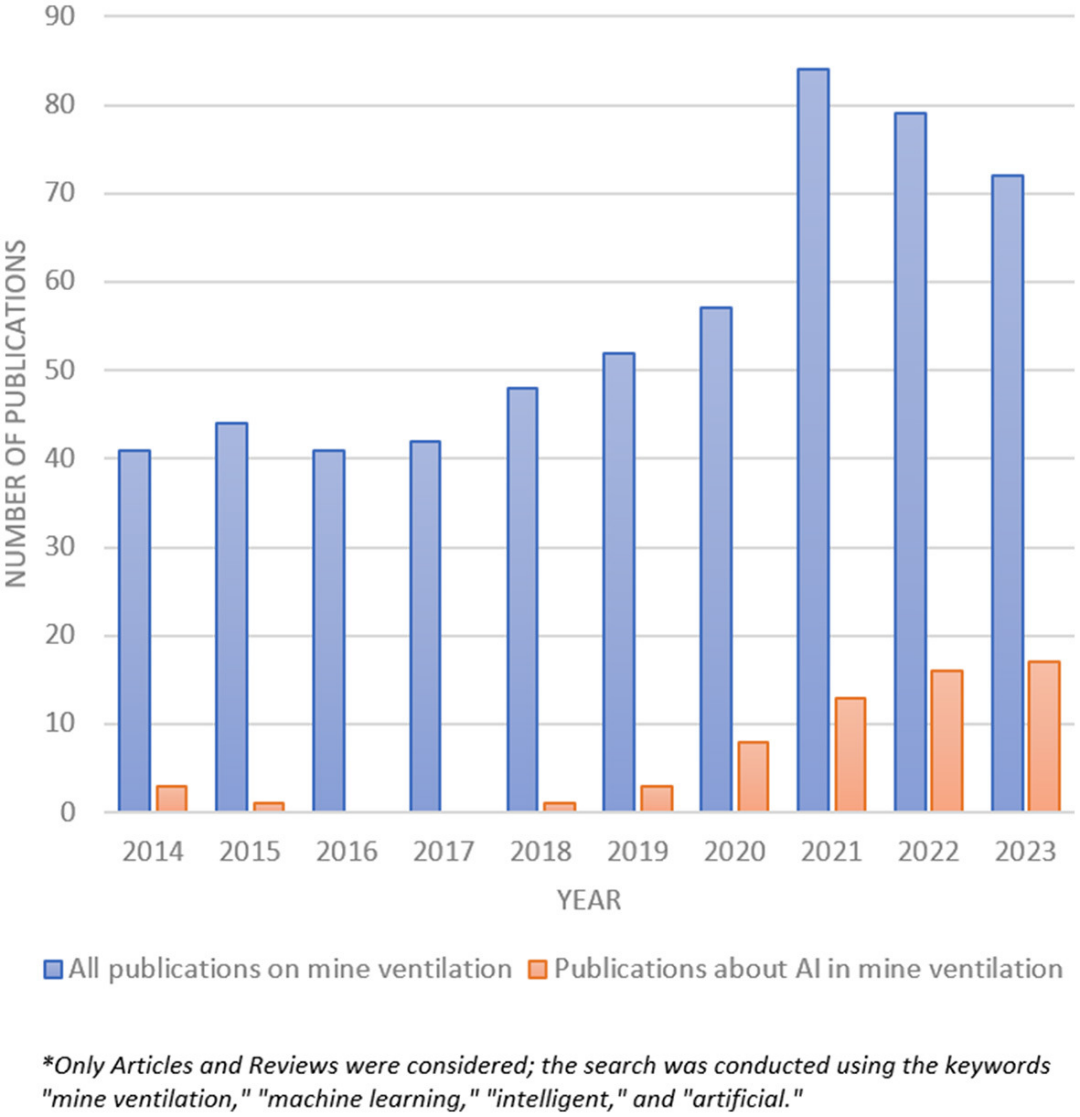
Time dynamics of the number of publications on mine ventilation in the period from 2014 to 2023.

Artificial intelligence (AI) has emerged as one such approach, offering the capability to discern hidden patterns, adapt to changing conditions, and provide forecasts amidst uncertainty. Consequently, AI is increasingly applied to mine ventilation tasks, as evidenced by our bibliometric analysis of publications in the past decade (see Figure 1), based on Scopus database.

Figure 1 illustrates that the proportion of publications discussing the application of AI within the total articles on mine ventilation has notably increased over the past four years, surpassing 20% by the end of 2023. Deep learning emerged as one of the frequently cited keywords in a cluster analysis of bibliographic works on mine ventilation spanning the past decade, as reported by Xue et al. (2024).

The utilization of AI in addressing mine ventilation challenges was examined in a review article by Hati (2021). The authors delineated various strategies for enhancing the energy efficiency of mine ventilation systems, explored the selection of components, and methods for intelligent prediction of airflow and contaminant concentrations. While the review provided a brief overview of AI technologies, including their general classification and advantages and disadvantages, it lacked an analysis of specific mine ventilation problems where AI has been effectively applied. Furthermore, there was no classification of AI technologies based on different areas of mine ventilation problems. We attribute this partly to the timing of the review, which predates a significant portion of AI-focused publications in mine ventilation, as indicated in Figure 1.

Another literature review by Shriwas and Pritchard (2020) addressed an essential application of AI technologies in mine ventilation monitoring and control. While the review did not directly focus on AI, it explored the concept of the Industrial Internet of Things (IIoT), emphasizing the need for long-term storage and advanced analysis of monitoring data to detect hidden trends and potential emergencies (Zhou et al., 2017).

Considering the aforementioned points and the rapid increase in the number of articles on AI in mine ventilation, an urgent task is to conduct a comprehensive literature review of AI technologies concerning mine ventilation issues. This review, while concise due to the current limited number of publications on AI in mine ventilation, aims to delineate the primary directions of AI implementation in mine ventilation and identify promising areas for AI application. We have identified four broad categories of mine ventilation problems that are currently being addressed using AI technologies. Subsequently, we discuss each application area sequentially, culminating in a comprehensive discussion of published research on mine ventilation utilizing AI.

## 2 Application areas of AI technologies

### 2.1 Mine ventilation monitoring systems

One of the main questions facing a mining engineer is how, based on the readings of individual measurements of air flows and air pressures in a real mine airway, to reconstruct a comprehensive depiction of the air flow distribution in a mathematical model. If the mine has a relatively small number of airways, then this can be achieved by measuring the air flow (Q) and pressure drop (ΔP) in almost every working, and subsequently calculating the resistance of each airway using the Equation (1) (Liu et al., 2022a):


(1) $$\begin{array}{l} R=\frac{H}{Q^{2}} \end{array}$$

However, for extensive ventilation networks with hundreds or thousands of branches, obtaining a complete picture of air flow distribution becomes challenging. This necessitates reconstructing air flow distribution in mines based on data from a limited number of manual or automatic measurements.

Liu et al. (2022b) studied optimal sensor placement for monitoring air distribution in mines, suggesting that equipping < 30% of tunnels with air velocity sensors ensures accurate air flow reconstruction if there are over 200 mine airways. Gao et al. (2018) employed a modified genetic algorithm (GA) to solve the inverse air distribution problem, determining mine airway resistance from air flow and pressure measurements. Liu (2023) used a GA-optimized neural network algorithm to assess mine ventilation system safety. Cao et al. (2024) applied deep reinforcement learning (DRL) to adjust mine airway resistances dynamically, showing smaller errors compared to other algorithms. This was demonstrated in a ventilation network with 153 branches. Ventilation resistance coefficient inversion has been explored by other researchers without AI (Li et al., 2019; Wu et al., 2024), but we omit these studies here.

Reconstructing air distribution in mines based on air velocity measurements is part of an intelligent mine monitoring system, considering mines within the IoT framework as cyber-physical systems. Kychkin and Nikolaev (2020) outlined four subsystems: physical objects, IoT network and computing infrastructure, digital twin, and human-machine interface. Jo and Khan (2018) proposed an IoT system for air quality monitoring, integrating pollutant assessment and forecasting features using an ANN model. Liu et al. (2020) integrated GIS and a transient ventilation network model to support real-time decision-making, envisioning an intelligent mine ventilation system.

Mining airway resistance is influenced by both well-formalized geometric parameters of the airway (such as cross-sectional area S, perimeter P, and length L) and poorly formalized parameters (including lining type and related parameters). The latter factor is typically implicitly considered in the air resistance coefficient α, from which R is derived:


(2) $$\begin{array}{l} R=\frac{\alpha PL}{S^{3}} \end{array}$$

In some cases, instead of α, other similar coefficients are used in the Equation (2) (McPherson, 2012; Amiri et al., 2020).

Determining α for mine airways with complex linings poses challenges, prompting researchers to turn to AI solutions. Zhao and Chen (2022) demonstrated SVM's effectiveness in modeling ventilation resistance coefficients based on mine airway geometry and lining parameters. For more intricate linings, Song et al. (2021) suggested using the PSO-SVM algorithm, known for its high accuracy, as a methodological guide.

Mine airway resistances change over time due to various factors (Skopintseva and Balovtsev, 2020). Such random changes in resistance are called resistance faults (Wang et al., 2023). These changes, whether short-term or gradual, can disrupt air distribution, posing risks of impurity buildup. Locating resistance faults is challenging and leads to active research in AI-based solutions.

Liu et al. (2022a, 2023) proposed resistance fault diagnostic models based on K-nearest neighbor (KNN), multilayer perceptron (MLP), SVM, and decision tree (DT). The authors showed high accuracy of these models in simulation and field studies. Wang et al. (2022) introduced an multi-label k-nearest neighbor (ML-KNN) model for rapid resistance failure identification in extensive ventilation networks. Subsequent work by Wang et al. (2023) presented a more sophisticated supervised learning model, combining DT, MLP, and ranking SVM, showcasing improved performance on complex networks. Zhao and Chen (2022) developed a fault scope library incorporating air volume and resistance relationships for diagnosing fault locations using SVM.

Cheng et al. (2014) and Cheng (2016) addressed a broader issue concerning reliability allocation in mine ventilation system design. The authors employed fuzzy mathematics and a Monte Carlo simulation approach to develop a model for scientifically allocating reliability practices.

### 2.2 Graphical analysis of ventilation networks

With the expansion and branching of mine ventilation networks, their graphical analysis also becomes more complex. The Ventilation Network Feature Graph (also known as a Q-H graph) offers a novel approach to directly and quantitatively represent the condition of a ventilation system and serves as an effective tool for studying complex ventilation networks (Xu and Tien, 1993). However, constructing such a graph requires a non-trivial analysis of the relationships between the elements of the ventilation network. For this reason, Jia et al. (2020) utilized GA to construct graphs that visually depict the main characteristics of the ventilation network, including air flows and pressure losses.

Initially, the Q-H plotting method faced challenges. For instance, when applied to a 3D ventilation network, the Q-H graph image was unavoidably segmented into rectangular blocks during the drawing process. However, Xie and Wang (2023) addressed this issue by refining the independent path sorting algorithm. They employed GA to expedite the creation of characteristic maps of ventilation networks on the Q-H axes.

3D reconstruction of mine airways is another important direction in the development of AI technologies in both mine ventilation and geotechnology (Ren et al., 2019; Du et al., 2023). Machine vision was utilized to process measurement data of the geometry of mine airways and mining equipment using various sensors: visual, inertial, LiDAR, and their combinations (Artan et al., 2011; Jiang et al., 2019; Zhai et al., 2020; Singh et al., 2023). This information can be further used to estimate changes in mine air resistance (Wong et al., 2011; Lavigne and Marshall, 2012; Watson and Marshall, 2018; Fahle et al., 2023).

In general, literature contains many works on the graphical methods for analyzing topologically complex mine ventilation networks (Maleki et al., 2018; Wu et al., 2019; Liu et al., 2022b; Bosikov et al., 2023), but we do not discuss these works here, since they are not use AI technologies.

### 2.3 Dynamics of gases and emergency ventilation

Mine ventilation, employing AI technologies, plays a crucial role in predicting harmful impurity release and emergency situations like fires. However, pinpointing emission locations and quantities can be challenging due to geological, geographical, and operational factors. Karacan and Goodman (2008) used principal component analysis and ANN to predict methane emission rates in longwall mines, aiding in selecting optimal degassing systems. Mathatho et al. (2019) developed an ANN model predicting methane concentration in coal mines based on microclimatic air parameters. Lin et al. (2022) analyzed gas emission monitoring data, predicting emissions in a dead-end face using SVM and GA for parameter optimization.

To a lesser extent, the literature provides examples of the implementation of AI to predict the generation and dynamics of dust in underground mines. Dust from blasting operations in open-pit mines has mainly been studied, wherein the dynamics of the dust cloud depend on numerous weather and technical factors that are challenging to formalize and accurately determine (Nagesha et al., 2016; Bakhtavar et al., 2021; Hosseini and Pourmirzaee, 2024).

Comfort and safety in mines depend not only on gases and dust but also on thermal factors (Cheng and Yang, 2012). Roy et al. (2022) employed GA to correlate environmental parameters with heat stress, albeit few studies focus on the thermal regime using AI. AI models, such as the PSO-SVR proposed by Deng et al. (2018), can predict coal spontaneous combustion temperatures based on gas concentrations in working areas. Ihsan et al. (2024a) applied a hybrid integrated numerical method and an ANFIS method to predict the wet bulb globe temperature.

Hong et al. (2022) utilized a supercomputer to simulate 1000 instances of a 3D mine tunnel fire under varying ventilation, thermal, and geometric conditions. They employed SVM, CART, RF, and ANN to predict fire dynamics, successfully forecasting backflow occurrence and smoke layer length. Basu et al. (2019) introduced a novel fuzzy logic system for underground coal mine fire hazard prediction, integrated into a wireless monitoring setup. Input parameters included air temperatures and impurity concentrations. Brodny et al. (2022) developed a similar system based on fuzzy logic and ANFIS to forecast methane accumulation hazards in a longwall.

Liu et al. (2021) adopted SVR to analyze shock wave propagation from methane explosions in mine ventilation networks. Their model, trained on numerically simulated airflow parameters, enables rapid assessment of explosion consequences.

### 2.4 Mine ventilation control systems

The mine ventilation system must not only be safe but also energy-efficient (de Vilhena Costa and Margarida da Silva, 2020; Ihsan et al., 2024b). The issue of selecting the optimal energy consumption parameters for the operation of main fans and ventilation doors is difficult to formalize for modern ventilation networks with a large number of air regulators. For such mines, finding the optimum can be very challenging (Wallace et al., 2015; Semin et al., 2020). To address this problem, Kashnikov and Levin (2017) proposed an Artificial Neural Network (ANN) model to enhance the performance of an algorithm for optimal ventilation control in a potash mine. Kashnikov and Kruglov (2022) applied fuzzy logic to determine fan influence zones under conditions of their dynamic change.

Huang and Liu (2022) laid out a theoretical basis for the intelligent design of mine ventilation. They demonstrated that an intelligent mine ventilation system should utilize full real-time monitoring data. Yang et al. (2023) introduced a method for remote intelligent air control based on machine learning, comprising three main components: fan frequency regulation, associated branch air resistance regulation, and their comprehensive integration. The authors employed five different models to predict the operating parameters of ventilation equipment with a given air volume. The results indicated that the Least Squares Support Vector Machine (LS-SVM) provides the most accurate predictions of target air parameters in the ventilation network, while maintaining a relatively short calculation time. These results were demonstrated on a laboratory bench of the mine consisting of 12 branches. Cheng et al. (2010) utilized fuzzy logic to optimize and evaluate the mine ventilation plan under conditions of a large number of varying parameters.

Hati and Kumar (2023) proposed a new algorithm that combines Adaptive Neural Fuzzy Interface System (ANFIS) and Genetic Algorithms (GA) to predict energy consumption and airflow in a mine ventilation system. This study compared the performance of the proposed algorithm with three other AI models: ANN, ANN-GA, and ANFIS.

Ihsan et al. (2024b) investigated AI-based Ventilation on Demand, which addresses both safety and energy efficiency concerns in mine ventilation. The authors suggested integrating real-time sensors, data, and ANFIS. However, the effectiveness of the system has only been assessed in laboratory experiments.

Wang et al. (2024) developed a non-linear optimization mathematical model of a mine ventilation network that minimizes total energy consumption for ventilation needs. They proposed a modified Sooty Tern Optimization Algorithm (STOA), belonging to the class of evolutionary algorithms, inspired by the behavior of gray terns during their long migrations.

## 3 Discussion

It is known that deterministic methods are more effective in the study of deterministic physical processes, while heuristic and intelligent methods demonstrate their efficacy in tasks where an explicit connection between the studied parameters and phenomena is not readily apparent. It is in this context that AI technologies are being integrated into mine ventilation systems today. Physical processes within mine atmospheres are well-understood by engineers and scientists and are typically modeled using deterministic approaches, such as solving systems of Kirchhoff equations to determine air distribution and solving differential equations for convective-diffusion transfer of heat and harmful impurities through mine workings (Olkhovsky et al., 2024).

However, the mine ventilation network is a complex system with numerous unpredictable and unaccounted factors. Analyzing these factors within deterministic frameworks often proves challenging or leads to failure, making heuristic approaches and machine learning methods more suitable. A summary of the AI technologies used today in mine ventilation is given in Table 1.

**Table 1:** Summary of AI technologies used in mine ventilation.

**Application areas**	**Specific problems**	**Applied models**	**References**
Mine ventilation monitoring systems	Solution of the inverse air distribution problem	GA	Gao et al., 2018; Liu et al., 2022b; Liu, 2023; Cao et al., 2024
	Modeling ventilation resistance coefficients	SVM, PSO-SVM	Song et al., 2021; Zhao and Chen, 2022;
	Identification of resistance faults	KNN, NL-KNN, MLP, SVM, ranking SVM, DT	Liu et al., 2022a, 2023; Wang et al., 2022, 2023; Zhao and Chen, 2022
	Reliability allocation	Fuzzy logic, Monte-Carlo	Cheng et al., 2014; Cheng, 2016
Graphical analysis of ventilation networks	Construction of ventilation network feature graph	GA	Jia et al., 2020; Xie and Wang, 2023
	3D visualization of mine airways	ANN	Ren et al., 2019; Chen et al., 2020
Dynamics of gases and emergency ventilation	Methane emission in mine airways	ANN, SVM-GA	Mathatho et al., 2019; Lin et al., 2022
	Calculation of heat stress	GA	Roy et al., 2022; Ihsan et al., 2024a
	Coal spontaneous combustion temperatures	PSO-SVR	Deng et al., 2018
	Fire dynamics in inclined airways	SVM, CART, RF, and ANN	Hong et al., 2022
	Methane accumulation in a longwall	fuzzy logic and ANFIS	Basu et al., 2019; Brodny et al., 2022
	Shock wave propagation from methane explosions	SVR	Liu et al., 2021
Mine ventilation control systems	Remote intelligent air control	ANN, LS-SVM	Kashnikov and Levin, 2017; Yang et al., 2023
	Minimizing energy consumption	ANN, ANFIS, ANFIS-GA, STOA	Hati and Kumar, 2023; Ihsan et al., 2024b; Wang et al., 2024

It is noteworthy that despite the achieved results, researchers have primarily applied these methods to ventilation networks with a relatively small number of branches (10–20). In rare cases, the number of branches may reach 100–200, which still falls short of fully meeting the needs of mine ventilation specialists. Many mine ventilation networks consist of thousands of branches (Semin and Levin, 2019). Hence, it is imperative to focus on studying the effectiveness of AI technologies for analyzing ventilation networks in large mines in the future.

Another interesting question is how many measuring stations are needed to correctly assess air distribution in the entire mine while accurately determining the location of resistance failures in mine airways. The answer to this question depends on the number of branches in the ventilation network and should also consider the topology features such as through-flow and U-tube ventilation layouts.

A change in the air resistance of mine airways is not the only unaccounted factor that can lead to alterations in air distribution within a mine ventilation network. Other possible reasons for observed deviations in measured air velocities at measuring stations could include thermal depression (Nikolaev and Klishin, 2021) and gas depression (Zhou and Wang, 2018). For example, unaccounted-for heat and gas emissions from mining equipment, as well as gas emissions from rock masses, can also significantly impact mine air distribution.

Calculations of unsteady transport of harmful impurities and propagation of shock waves on deterministic models of ventilation networks are time-consuming due to the need for greatly reduced cell sizes and time steps. Therefore, it is sometimes appropriate to use heuristic approaches to solve deterministic problems in order to conserve computing resources. The most computationally expensive problems include calculating fires in mining systems (Hong et al., 2022) and propagating shock waves (Liu et al., 2021). Consequently, scientists are currently working on developing faster methods for performing these calculations. While the proposed models to date are still far from perfect due to several unaccounted factors and errors, this approach is likely to receive active development in the future. This is because in emergency mine ventilation situations, the speed of calculations is crucial for quickly finding effective solutions (Onifade, 2021).

Another promising area for utilizing AI in mine ventilation may be determining the shock losses of mine airways when calculating air distribution. Semin and Levin (2023) highlighted the importance of shock loss factors, especially in airways with large cross-sections. However, existing approaches to calculating shock losses lack both sufficient accuracy and versatility, particularly in their applicability to various types of mine airway junctions. The use of surrogate modeling could facilitate the selection of an approximating function for shock loss factors based on mine airway parameters, potentially providing accurate solutions across a wide range of mine airway junctions.

## 4 Conclusion

We anticipate that the utilization of intelligent methods for solving mine ventilation problems will increase in the future. This trend will be propelled by advancements in computing power and the emergence of new, more efficient AI technologies. Recognizing the existing gaps, AI will be progressively deployed across a broader spectrum of mine ventilation challenges. Concurrently, deterministic approaches will persist in analyzing mine ventilation systems, fostering a growing integration with heuristic and machine learning methodologies.

## Author contributions

MS: Conceptualization, Data curation, Formal analysis, Funding acquisition, Investigation, Methodology, Project administration, Resources, Software, Supervision, Validation, Visualization, Writing – original draft. DK: Conceptualization, Funding acquisition, Investigation, Writing – review & editing.
